# Effect of Coffee Grounds Content on Properties of PHBV Biocomposites Compared to Similar Composites with Other Fillers

**DOI:** 10.3390/polym17060764

**Published:** 2025-03-14

**Authors:** Grzegorz Janowski, Wiesław Frącz, Łukasz Bąk, Janusz W. Sikora, Adam Tomczyk, Grażyna Mrówka-Nowotnik, Beata Mossety-Leszczak

**Affiliations:** 1 Department of Materials Forming and Processing, Rzeszow University of Technology, Powstancow Warszawy 8, 35-959 Rzeszow, Poland; lbak@prz.edu.pl; 2 Department of Technology and Polymer Processing, Lublin University of Technology, Nadbystrzycka 36, 20-618 Lublin, Poland; janusz.sikora@pollub.pl; 3 Department of Mechanics and Applied Computer Science, Faculty of Mechanical Engineering, Białystok University of Technology, Wiejska 45C Str., 15-351 Białystok, Poland; a.tomczyk@pb.edu.pl; 4 Department of Material Science, Rzeszow University of Technology, Al. Powstańców Warszawy 12, 35-959 Rzeszow, Poland; mrowka@prz.edu.pl; 5 Department of Industrial and Materials Chemistry, Rzeszow University of Technology, al. Powstańców Warszawy 6, 35-959 Rzeszów, Poland; mossety@prz.edu.pl

**Keywords:** spent coffee grounds (SCG), injection molding technology, green polymers, wood polymer composites, hemp polymer composites

## Abstract

Spent coffee grounds (SCG) have potential as a sustainable bio-filler in poly(3-hydroxybutyrate-co-3-hydroxyvalerate) (PHBV) composites, offering an environmentally friendly approach to waste utilization. This study investigated the effect of SCG content on the mechanical, thermal, and morphological properties of PHBV biocomposites and compared them with those of composites containing wood flour and hemp fibers. The biocomposites were fabricated via extrusion and injection molding, followed by the characterization of their mechanical performance, thermal behavior, and microstructure. The results indicated that SCG increased the stiffness of PHBV but did not enhance its tensile strength due to the weak interfacial adhesion between the filler and matrix. Unlike other lignocellulosic fillers, SCG requires lower processing temperatures, which is advantageous for thermally sensitive applications. SEM analysis revealed well-dispersed SCG particles at low concentrations, but visible aggregation and interfacial voids at higher loadings. While SCG serves as an effective and cost-efficient filler for improving the stiffness of PHBV, it does not reinforce the material in the conventional sense.

## 1. Introduction

The management of polymer waste remains a critical environmental challenge, as the most widely used polymers are non-biodegradable. The increasing production and consumption of polymer-based materials necessitates the development of responsible waste management strategies. In 2020, of the nearly 30 million tons of post-consumer polymer waste collected, only 35% was recycled, 42% was used for energy recovery, and 23% was landfilled [1]. Meanwhile, the share of environmentally friendly bioplastics remains marginal at just 2.4% [2].

A promising approach to reducing polymer waste is the development of biopolymers, which are derived from natural/renewable sources and are biodegradable. Among them, polyhydroxyalkanoates (PHAs) have gained attention due to their microbial origin and complete biodegradability. A notable member of this group is poly(3-hydroxybutyrate-co-3-hydroxyvalerate) (PHBV), which can be synthesized by bacteria as a storage material [3]. PHBV exhibits properties suitable for various applications; however, its widespread commercialization is hindered by high production costs and processing limitations. Its high brittleness and lower temperature difference between the melting and degradation temperatures compared to those of commonly used polyolefins limit its industrial applications [4,5,6,7,8]. One strategy to mitigate these limitations is the incorporation of natural fillers into PHBV-based biocomposites. This approach can reduce production costs while preserving the material’s biodegradability [9,10]. Among the potential fillers, spent coffee grounds (SCG) stand out due to their high lignin content, which includes fatty groups that can influence the processing behavior of PHBV composites.

The consumption of coffee generates significant amounts of spent coffee grounds (SCG), which can be repurposed in various industries, including in polymer processing. Due to their natural origin, SCG are particularly suitable as fillers for biodegradable polymer composites. One potential application is the incorporation of SCG into poly(3-hydroxybutyrate-co-3-hydroxyvalerate) (PHBV) matrices, which could enhance the commercial viability of PHBV-based biocomposites while promoting sustainable waste management.

The justification for utilizing SCG as a filler is multifaceted. On average, coffee consumption results in approximately 10 g SCG per serving. A typical Polish consumer drinks around 3 kg of coffee annually, which is equivalent to roughly one cup per day. Over the past decade, coffee consumption in Poland has increased by more than 80%, leading to substantial amounts of waste generation. For instance, 100 coffee shop chains collectively produce around 180 tons of SCG per year, contributing to an estimated annual national total of 120,000 tons. The Polish coffee market, which encompasses coffee beans, ground coffee, capsules, and instant coffee, was valued at approximately PLN 5.27 billion in 2013 [11,12,13,14].

The integration of SCG into PHBV composites aligns with circular economy principles by repurposing organic waste and reducing the reliance on conventional fillers. Further research is required to optimize SCG-polymer interactions and assess the mechanical, thermal, and biodegradation properties of the resulting composites.

Spent coffee grounds (SCG) are increasingly used as fillers in polymer composites, including polylactide (PLA, PLLA), bio-based polyethylene (Bio-PE), and bio-polyamides (Bio-PA), particularly in packaging and fast food applications [15,16,17]. Their natural origin makes them compatible with biopolymer matrices; however, their susceptibility to fungal degradation remains a challenge.

The incorporation of coffee waste into polymer matrices has been widely explored. Huang et al. [18] investigated coffee husk fiber (CHF) as a filler in high-density polyethylene (HDPE), finding similar properties to wood fiber (WF), with thermal stability up to 210 °C. The addition of maleic anhydride polyethylene (MAPE) improved the interfacial adhesion between the hydrophilic filler and hydrophobic polymer. The addition of CHF (40–70%) enhanced the mechanical and thermal properties but increased water absorption.

Cestari et al. [19] studied HDPE composites with coffee grounds (10–60%), showing minimal impact on melting temperature and crystallinity, with comparable compressive modulus to pure HDPE. Baek et al. [9] reported a decline in mechanical performance of PLA composites as SCG content increased (e.g., tensile strength from 60.1 MPa for pure PLA to 22.4 MPa at 40% filler).

Other studies have demonstrated varying effects of coffee husks on polymer properties. For HDPE, low concentrations (1–2%) enhanced thermo-oxidative stability (oxidation induction time increased by 150%) while maintaining tensile strength; however, excessive loading (>5%) led to reduced ductility due to filler agglomeration [20]. SCG also showed potential as a sustainable alternative to carbon black in nitrile rubber, improving elasticity (+20% elongation at break) and thermal stability while reducing dielectric conductivity [21].

In polypropylene (PP), SCG influenced the mechanical properties depending on the polymer type. Homopolymer PP composites exhibited stable mechanical performance up to 20% filler content, whereas copolymer PP showed a continuous decline in strength and impact resistance with increasing SCG content. SEM analysis revealed micropores at the filler-matrix interface, which weakened the mechanical properties [22]. Zarrinbakhsh [23] compared coffee husks and SCG as PP reinforcements and found that coffee husks provided superior thermal stability and mechanical reinforcement due to their fibrous structure, whereas SCG, with its higher fat content, had a plasticizing effect.

Kumar et al. [24] examined coffee husk fractions (150–425 µm) in PLA and bio-PBS matrices, demonstrating improved mechanical properties in bio-PBS composites with 10% fine-particle filler. While PLA composites showed slight crystallinity growth, bio-PBS crystallinity decreased due to the catalytic effects of coffee husk metal content. Bio-PBS composites with smaller filler particles exhibited better thermal stability and processability than PLA-based composites did.

Some researchers have already made some attempts to produce biocomposites from spent coffee grounds (SCG) based on poly(hydroxyalkanoates) (PHA).

Tang et al. [25] investigated the enhancement of SCG–PHA interfacial compatibility through three surface modification methods: alkali treatment, silane treatment, and their combination. The results showed that all treatments improved the mechanical, thermal, and water resistance properties of the composites, with the alkali/silane combination treatment being the most effective one. Increases in tensile strength, flexural strength, and elongation at break indicated better fiber-matrix adhesion. FTIR, SEM, and XRD analyses confirmed the increased surface roughness, higher crystallinity, and stronger chemical interactions after modification. These findings suggest that properly modified SCG can serve as an effective and sustainable filler in biodegradable PHA-based composites, enhancing their potential applications in packaging and other biopolymer-based industries.

Alharbi et al. [26] conducted a study on the use of spent coffee grounds (SCG) as a microfiller in poly(3-hydroxybutyrate-co-3-hydroxyvalerate) (PHBV) biocomposites, aiming to enhance their mechanical and thermal properties for biodegradable packaging applications. PHBV/SCG composites were produced using twin-screw extrusion and hot pressing, with SCG content varying at 1%, 3%, 5%, and 7%. The results showed that at an optimal SCG content of 5%, the composites exhibited improved mechanical strength, wettability, and morphological structure, as confirmed by FE-SEM and AFM analyses. However, further increasing the SCG content to 7% led to particle agglomeration, resulting in a decline in the mechanical properties. TGA analysis indicated that the PHBV/SCG composites maintained thermal stability up to 278 °C, while DSC tests showed minimal changes in the melting temperature, suggesting that SCG addition did not significantly affect the polymer crystallization process. These findings highlight the potential of spent coffee grounds as a sustainable reinforcing filler for biopolymers, while emphasizing the need to optimize their concentration to avoid the adverse effects of agglomeration.

Reis et al. [27] conducted a study on the use of lignocellulosic waste particles from the coffee industry (coffee husk—CH and coffee parchment—CP) as reinforcing fillers in poly(hydroxybutyrate) (PHB)-based biocomposites. The impact of filler type and content (0%, 10%, and 20%) on the mechanical, thermal, microstructural, and water absorption properties was analyzed. The results showed that the addition of CH and CP increased the thermal degradation temperature of the composites, with a higher CP content leading to a reduction in the PHB crystallinity. Mechanically, the addition of 10% CP did not significantly affect tensile strength or impact resistance, while a 20% CP content resulted in improved mechanical properties, suggesting enhanced polymer matrix reinforcement. However, increasing the filler content also led to higher water absorption, which is a critical factor for the material’s applications. The findings indicate that coffee waste can serve as an effective and sustainable filler for biodegradable PHB composites, although optimizing the filler content is necessary to balance the improved mechanical properties with material stability.

Boey et al. [28] investigated the effect of spent coffee grounds (SCG) content, matrix ratio, and biological treatment of SCG on the properties of poly(hydroxybutyrate) (PHB) and poly(lactic acid) (PLA) composites. This study aimed to develop and characterize SCG-reinforced biocomposites in terms of their mechanical, morphological, and thermal properties. The results showed that increasing SCG content up to 40% led to particle agglomeration and increased porosity, negatively affecting tensile strength. The optimal PHB/PLA ratio was 50/50 with 20% SCG, providing the best balance between mechanical and processing properties. Additionally, the biological treatment of SCG using Phanerochaete chrysosporium CK01 and Aspergillus niger DWA8 improved the interfacial adhesion and particle distribution in the matrix, as confirmed by SEM analysis. This study demonstrated that biologically treated SCG enhances compatibility with the polymer matrix, improving its mechanical properties and reducing structural defects, thereby highlighting its potential as a sustainable filler in PHB/PLA biocomposites.

Zhao et al. [29] conducted a study on the production of poly(3-hydroxybutyrate-co-3-hydroxyvalerate) (PHBV)-based biocomposites with the addition of spent coffee grounds (SCG) The authors applied a reactive extrusion method without the need for prior SCG modification. This study analyzed the effects of SCG content (10–30%) and various processing additives (peroxide, coagent, and chain extender) on the morphological, thermal, mechanical, and water absorption properties of PHBV/SCG composites. The results showed that the addition of SCG reduced the PHBV crystallinity (by 3.8–6.8%), degradation temperature (by 6.2–8.2%), and tensile strain (by 33–43%) while having no significant effect on the tensile strength and Young’s modulus. Meanwhile, the processing additives reduced the melting temperature (by 4.5 °C), crystallinity (by 8.4%), and Young’s modulus (by 17%), while increasing the degradation temperature (by 12.5%). Additionally, SCG significantly enhanced PHBV’s water absorption ability (by 250%), and the addition of peroxide further boosted this effect to 320%.

Presnell [30] conducted a study on the development of poly(3-hydroxybutyrate-co-3-hydroxyvalerate) (PHBV)-based biocomposites with the addition of spent coffee grounds (SCG) to create biodegradable materials with improved mechanical, thermal, and water absorption properties. Eight sample variants were prepared, including PHBV with 10%, 20%, and 30% SCG, as well as processing additives such as peroxides, coagents, and chain extenders. The results showed that SCG increased the crystallinity of PHBV, with the 30% SCG sample reaching 58.7% crystallinity compared to 59.6% for pure PHBV, suggesting its structural stability. Additionally, SCG slightly increased water loss (~0.3%), indicating the composite’s ability to retain moisture, which is beneficial for greenhouse planter applications. TGA tests revealed that increasing SCG content lowered the degradation temperature, while samples with higher SCG content retained more residual mass after degradation, suggesting their potential for applications requiring thermal resistance. Mechanical tests indicated that SCG had no significant impact on tensile strength; however, samples with additives (PHBV/SCG10/P, PHBV/SCG10/P/C, and PHBV/SCG10/P/C/S) exhibited lower tensile strength. The findings suggest that SCG can serve as a sustainable filler for PHBV biocomposites, and further optimization of the SCG content may enhance the mechanical strength and biodegradability of materials for packaging and agricultural applications.

Against the backdrop of existing research on PHBV composites filled with spent coffee grounds (SCG), this study aims to expand the understanding of their properties and potential applications in the biodegradable plastics industry. Previous studies have examined various aspects of these composites, including the impact of chemical treatment on interfacial compatibility [25], optimal SCG content for mechanical and thermal properties [26], use of different coffee industry waste fractions [27], effectiveness of biological modification to improve SCG-polymer interactions [28], and application of reactive extrusion as an alternative to costly pretreatment processes [29].

This study aims to complement these investigations by comparing the properties of PHBV-based biocomposites filled with SCG to those containing other natural fillers, such as wood flour and hemp fibers [7,31,32]. This comparative analysis may provide insight into whether SCG can serve as a viable alternative to conventional fillers in biopolymers and highlight its potential advantages and limitations in terms of mechanical strength, thermal stability, and microstructure. Furthermore, this research focuses on the influence of SCG on the processing parameters of PHBV during extrusion and injection molding, which could help identify the key factors determining the optimal processing conditions for these composites.

The findings of this study could offer valuable insights into the feasibility of SCG as an alternative, sustainable filler in biodegradable polymer composites, particularly in the context of its potential commercialization. Utilizing coffee waste as a secondary raw material in biopolymers could contribute to enhancing sustainability in the biodegradable materials sector, reducing production costs, and expanding the range of applications for PHBV-based biocomposites.

## 2. Materials and Methods

### 2.1. Materials

In this research, PHBV, under the trade name Enmat Y1000 and in the form of a powder, was used as the polymer matrix [8]. This biopolymer was produced by the company Tianan Biopolymer (Ningbo, China). The molar proportion of 3-hydroxyvaleric acid (HV) was 8%, the density of the biopolymer was 1250 kg/m^3^, and the softening point was in the range of 165 °C to 175 °C.

Spent coffee ground (SCG) was used as a filler in the polymer matrix. The produced biocomposite contained coffee grounds with mass fractions of 15, 30, and 45%. The SCG was collected from restaurants where the primary type of coffee was Jacobs Kronung (Jacobs Douwe Egberts, Amsterdam, The Netherlands) and ground coffee. The grounds were dried for 24 h at 60 °C and stored in dry rooms before processing. Spent coffee grounds (SCGs) had an average size of about 10–300 µm (assessed on the basis of own research). For comparison purposes, the filler and wood flour had a particle distribution in the range of 70–150 µm [33]. Hemp fibers were long fibers with a high aspect ratio of 10, with an average length of 1 mm, and a diameter of about 100 µm [31,32].

### 2.2. The Manufacturing Process

The research procedure presented in this paper included the production of a biocomposite in the extrusion process, injection molding of the molded piece with the original processing parameters (for a new material), and determination of the mechanical and functional properties and shrinkage of the produced samples.

The PHBV and SCG were manually mixed to ensure the highest possible uniformity in composition. The mixture underwent a drying process before single-screw extrusion. Drying was conducted at 90 °C for 6 h using a DZ-2BC laboratory dryer (Chemland, Stargard, Poland). This step was crucial for reducing the presence of air bubbles in the extrudate cross-sections.

A single-screw extruder (ZAMAK EHP 25E, ZAMAK-Mercator, Skawina, Poland) was used to extrude the biocomposites. The analysis of the extrusion process of biocomposites with different types of natural fillers (Table 1) in terms of heat indicated significant differences in the processing temperature for the produced biocomposites, especially for biocomposites with SCG in order to obtain pomace of similar quality. The granulation of the material was performed in parallel with the extrusion process using a ZAMAK G16/32-II granulator (ZAMAK-Mercator, Skawina, Poland).

The specimens for testing the mechanical properties were produced using a Dr Boy 55E injection molding machine (BOY Maschines Inc., Exton, PA, USA). In this research, an injection mold with a specialized insert was used to produce paddle-shaped samples for uniaxial tensile testing, in accordance with the EN ISO 527-1 standard. In Table 2 and Table 3 the processing parameters of the injection molding process (temperature, pressure, velocity) of biocomposites with tested fillers and pure PHBV are presented.

### 2.3. Methods

Microscopic images of the geometry and dimensions of SCG were captured using a Nikon LV100 (Nikon Company, Minato, Japan) optical microscope at a magnification of 50×. Microstructural images of the sample fractures were captured using a HITACHI S-3400N (Hitachi, Tokyo, Japan) scanning electron microscope (SEM) at magnifications of 125×, 500×, and 3000×. Microscopic and SEM micrographs were obtained for five samples of each type, and representative sample images are presented in this paper.

Thermal analyses of the biocomposite were performed using a Mettler-Toledo DSC differential scanning calorimeter (Mettler-Toledo, Greifensee, Switzerland). The thermal properties of the analyzed samples were measured using a Mettler-Toledo DSC1 differential scanning calorimeter cooled with liquid nitrogen. The DSC device was calibrated using zinc and indium. The analyses were performed in 40 µL aluminum pans, hermetically sealed with a lid with a small hole, in a nitrogen atmosphere (flow rate 60 mL·min^−1^), in accordance with the standard EN ISO 11357-1 [34]. The DSC measurements were carried out at a heating rate of 10 K·min^−1^, −50 °C to 250 °C.

A Zwick Z030 (Zwick/Roell, Ulm, Germany) testing machine was used for tests to determine the mechanical properties of the obtained composites. A uniaxial tensile test was carried out in accordance with the EN ISO 527-1 [35] standard for moldings with a “dumbbell” geometry.

The hardness of the biocomposites was tested using the ball indentation method in accordance with EN ISO 2039-1 [36]. The hardness was tested in two areas of each sample: A (the central part of the sample between the grips) and B (the area near the grips) using a Zwick 3106 hardness tester (Zwick/Roell, Ulm, Germany).

The biocomposite samples were subjected to impact tensile testing. The impact tensile strength was determined according to the EN ISO 8256 [37] standard using a CAEST 9050 pendulum hammer (Instron, Norwood, MA, USA).

The shrinkage of the “dumbbell” geometry moldings was tested based on the EN ISO 294-4 [38] standard.

Uniaxial tensile, impact tensile strength, hardness, and shrinkage tests were performed on 10 samples of each type of material. For the obtained results (for 10 tested samples), the mean value and standard deviation were calculated for each material tested.

## 3. Results and Discussion

### 3.1. Assessment of Processing Capabilities

For example, in the case of a 15% coffee content in the composite, higher temperature values were used in the individual heating zones compared to pure PHBV [7]: 175 °C in the head, 170 °C in zone 3, 160 °C in zone 2, and 150 °C in zone 1, with the hopper zone having a temperature of 35 °C and the screw speed being 100 rpm. These temperature settings indicate the need for intensive heating to enable uniform melting and proper dispersion of coffee particles in the PHBV matrix. Coffee as a filler requires a relatively high temperature to ensure effective homogenization of the material, which is important for process stability and the quality of the final product.

When the coffee content was increased to 30%, the temperatures were reduced, especially in the head heating zones and the remaining heating zones, where the head temperature was 165 °C, and in the remaining zones the temperatures were reduced by about 5 °C compared to the value at 15% coffee content in order to obtain a pomace of similar quality as for other extruded materials [7,31,32]. The lower temperature is also beneficial because it reduces the risk of thermal degradation of coffee components, which may have a positive effect on the mechanical properties and stability of the biocomposite.

In the case of 45% coffee content, the temperature in all heating zones was reduced, with the head temperature being 157 °C and in the subsequent heating zones 155 °C, 150 °C, and 148 °C, respectively, while the hopper zone had a temperature of 33 °C. This temperature reduction was related to the need to avoid overheating the coffee. This, in turn, would negatively affect the physical and mechanical properties of the material. When comparing the results for biocomposites with SCG and fillers: wood flour and hemp fibers [7,31,32], clear differences in the required processing temperatures were observed.

For wood flour composites, both at 15% and 30% content, the temperature in the heating zones was relatively high, reaching 180 °C for the head. Hemp fiber composites at 15% content showed similar thermal characteristics to those of coffee ground composites; however, at higher contents, 30% and 45%, it was necessary to use a higher temperature, reaching 180 °C in the head.

When analyzing the injection molding process of composites containing different types of natural fillers, special attention was paid to the parameters of the composites with the coffee filler. Based on Table 2, certain relationships can be observed between the content of coffee filler and the required processing temperature and mold temperature.

For the composites with SCG as a filler content of 15%, the processing temperature was set at 183 °C, and the mold temperature was 80 °C. The high processing temperature, compared to that of pure PHBV (167 °C), resulted from the need to evenly distribute the coffee in the polymer matrix. A mold temperature of 80 °C, which is higher than that of PHBV (60 °C), favors the slow cooling of the molded part, which can prevent internal stresses and improve the mechanical properties of the product.

At 30% coffee content, the processing temperature was reduced to 175 °C, but the mold temperature was maintained at 80 °C. At the same time, the constant mold temperature of 80 °C provides optimal cooling conditions for the material, which allows obtaining a molded part with good mechanical properties without excessive risk of deformation.

For composites with 45% coffee content, the processing temperature was further reduced to 170 °C, while the mold temperature remained at 80 °C. The high coffee content required further reduction in the processing temperature, which resulted from the intensive impact of SCG on the PHBV polymer matrix, which became more susceptible to plasticization. Maintaining a constant mold temperature allows slow and controlled cooling, which is particularly important with such a high filler content since too rapid cooling could lead to stress formation and weakening of the molded part structure.

Comparing these results with those of the fillers wood flour and hemp fibers, it can be seen that the produced biocomposites require different temperature settings. For composites with wood flour at 15% content, the plastic temperature was 170 °C and the mold temperature was 65 °C, which is lower than that for SCG. At 30% and 45% content of wood flour, the processing temperature was increased to 175 °C and 180 °C, and the mold temperature to 85 °C and 90 °C, respectively. These higher values suggest that wood flour requires higher temperatures for higher content to ensure proper processing, homogenization, and filling of the molding cavity.

Hemp fibers, on the other hand, require even higher plastic and mold temperatures. For 15% fiber content, the plastic temperature was the same as that for pure PHBV (167 °C), and the mold temperature was 60 °C, similar to that of PHBV without filler. However, at 30% and 45% hemp fiber content, the processing temperature was increased to 185 °C and 190°C, respectively, and the mold temperature to 85 °C and 90 °C.

### 3.2. Spent Coffee Ground Geometry Assessment

**Figure 1:**
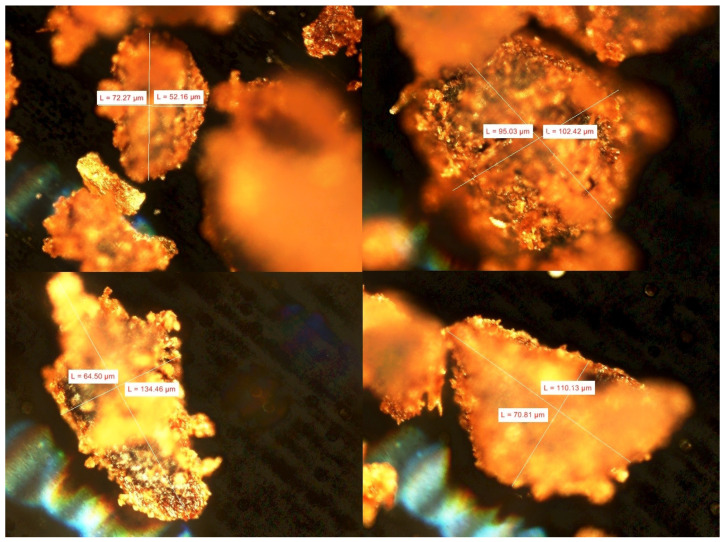
Examples of the geometry and size of SCG (magnification 50×).

Photographs of the dried SCG after use are shown in Figure 1. The particle sizes of the SCG, which range from about 10 to 300 μm, can affect their uniform distribution in the polymer. However, excessive size diversity can weaken the structure, especially if larger particles concentrate in one area, causing defects in the material. The optimal concentration of grounds as fillers depends on the requirements for the biocomposite properties.

### 3.3. An Analysis of Thermal Properties of Biocomposite

The measurements with the DSC method required the application of EN ISO 11357 standards. According to them, it is not necessary to repeat the analyses. However, the measurements for each sample under the used conditions were repeated two times, obtaining a very high repeatability. Therefore, no statistical analysis was performed.

**Figure 2:**
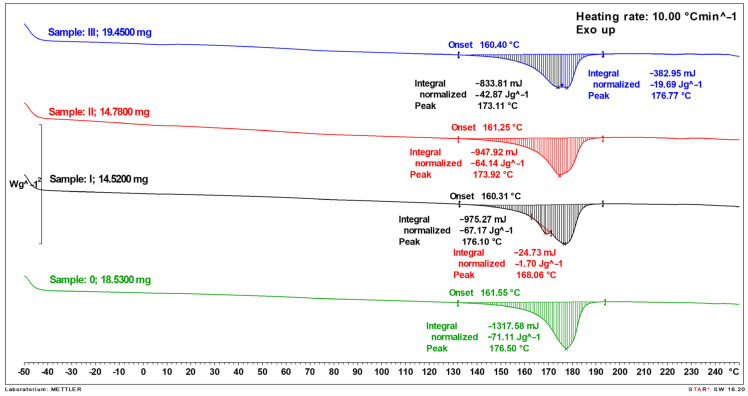
Heat flow rate dependence on temperature (DSC curves) for PHBV—SPC composites: sample 0—pure PHBV (powder), sample I—composite with 15 wt.% filler, sample II composite with 30 wt.% filler, Sample III—composite with 45 wt.% filler.

The DSC thermal curves of the composites with variable filler content in the form of SCG are presented in Figure 2. For comparison, a DSC curve was also recorded for the pure PHBV polymer (Sample 0). For each of the samples, the enthalpy change was determined, among other things, equal to the amount of heat needed to melt the sample, which is directly proportional to the amount of crystalline phase. The sample marked as Sample III contained the most filler (45%), and for this sample, the total enthalpy of melting expressed in J per gram of sample (not pure polymer) was the lowest (Figure 2). This is due to the fact that in this sample, the share of polymer and crystalline phase, the endothermic melting peak of which is recorded, is reduced by almost half. Subsequently, for sample II, which contained 30% filler, the enthalpy was higher compared to that of Sample III, while for the sample with the addition of this modifier in the amount of 15%, it was similar to that of the pure, reference polymer.

The fact that the data on the endothermic melting enthalpy shown in Figure 2 do not refer only to the polymer matrix, to which this transformation applies, essentially distorting the information on the content of the crystalline phase in the polymer. These data, after recalculation only for the polymer (Sample 0—100%, Sample I—85%, Sample II—70%, and Sample III—55%), together with other parameters resulting from the DSC analysis, are listed in Table 4. After recalculation, the values of the corrected enthalpy, which is related only to the polymer part in the composites, clearly indicated that the amount of the crystalline phase, which is directly proportional to the melting enthalpy, increases with an increase in the amount of filler. This relationship clearly suggests the nucleating nature of the filler used.

A slight change in the shape of the peak attributed to the melting of the crystalline phase was also observed. For the pure polymer (sample 0), no additional signal or peak deformation was visible, while for the sample with 15% filler (Sample I), an additional small signal appeared with a minimum at approx. 168 °C, for the sample with 30% SCG (Sample II), a deformation (“bulge”) was recorded above approx. 175 °C, which was visible in the form of a clear signal, such as the second peak minimum at 176.8 °C on the DSC curve of the sample with 45% filler (Sample III). This behavior of the tested samples indicates that the addition of SCG has a small effect on the thermal properties of the compactness and critical phase of the polymer matrix, slightly modifying the form and morphology of the crystals. The appearance of a different crystalline form (differences in the shape of the melting peak on the DSC curve between the pure polymer and the sample with the filler, as discussed above) is probably due to the addition of the filler and its amount. The crystalline form depends on the crystallization method after melting, and the addition of the filler can act, for example, as a nucleant or can change the morphology of the polymer crystallites in the composite.

The studies also showed that the addition of the filler did not affect the change in the melting temperature range. The beginning of this transformation for all samples was approximately 161 °C, which indicated that there was no degradation of the polymer during the preparation of the composites and their processing. It was also found that the melting range of the crystalline phase of the polymer (ab. 161–185 °C) in the composites does not change significantly, regardless of the amount of filler added.

### 3.4. Evaluation of Mechanical Properties in the Uniaxial Tensile Test

**Figure 3:**
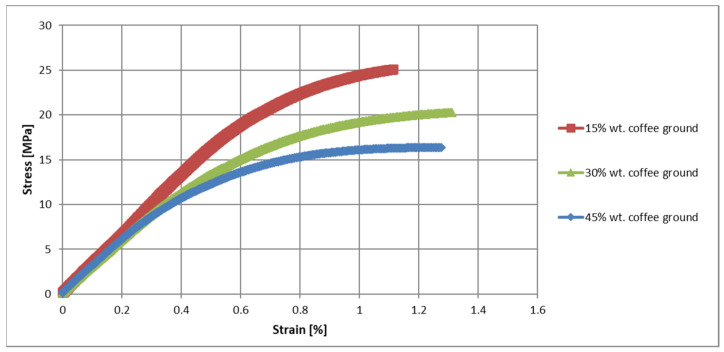
Stress-strain characteristics of PHBV-coffee grounds.

The analysis of the mechanical properties of the composites with SCG indicated a significant effect of the presence and content of grounds on the strength parameters of the composite (Figure 3, Table 5). At this stage, it is worth comparing the results with information from the literature for similar materials with a PHBV matrix or a pure polymer matrix [7,31,32]. Pure PHBV has a modulus of elasticity of 2617 MPa and a tensile strength of 35 MPa, with a maximum deformation of about 4%. The introduction of SCG caused noticeable changes—already at 15% filler content, the modulus of elasticity increased to 3308 MPa, which indicated an increase in the stiffness of the material. The growth in the modulus of elasticity can be explained by the presence of irregular, strongly jagged coffee particles which disperse in the matrix and limit its ability to deform, which increases the stiffness of the composite. As a filler with complex morphology, SCG increases the stiffness of the composite by creating local blockades for matrix deformation. However, their ability to strengthen decreases with increasing content—for 30% and 45% of grounds, we observe the modulus of elasticity at the level of 3085 MPa and 3092 MPa, respectively, which indicates reaching the limit of the stiffening effect of spent coffee grounds in the matrix. We compared our observations with those found in the literature—for example, Mohanty and Nayak [39] observed that for PP composites with jute fibers, the mechanical properties improved and reached a maximum value at 30%. A similar trend was also observed by Singh and Mohanty [40], where the maximum flexural strength was obtained for a composite containing 30% filler (composites were tested in the range of 10% to 40% filler content). Related information can also be found in the review paper by Thomason and Rudeiros-Fernández [41]. In contrast, Reis and co-authors, observed a decrease in Young’s modulus with the addition and increasein the content of SCG [27].

However, the tensile strength of the composites with SCG was lower than that of pure PHBV, and decreased with increasing grounds content, which suggested that higher filler content led to the formation of structural defects. At 15% ground content, the tensile strength was 23 MPa, which was noticeably lower than that of the pure polymer, and at 45% ground content, this value dropped to 16 MPa. The reduction in tensile strength may be the result of weaker adhesion between SCG and the matrix and the formation of areas of reduced cohesion, which is characteristic of irregularly shaped fillers. SCG, due to their porous structure, may not fully bond with the matrix, which results in local weakening and energy dissipation in areas with lower cohesion. Additionally, SCG particles are irregular and have a high specific surface area, which can lead to the formation of microvoids during composite processing—these micropores become points of stress concentration, which reduces the strength of the material under the influence of mechanical loads. We support our observations with reports from other researchers: Reis et al. [27] found that the addition of 20% SCG to PHB reduced the tensile strength due to poor adhesion and defects in the material. Similar results have been reported for other biocomposites—for example, Mysiukiewicz et al. [42] demonstrated a decrease in the tensile strength of PP with an increasing share of aluminum powder, despite an increase in stiffness. As can be seen, the observed phenomena are consistent with the literature describing fillers with a low aspect ratio.

The chemical composition and morphology of SCG significantly impact the mechanical properties of composites, especially at higher filler contents. SCG contain a relatively small amount of durable cellulose fibers (~10%) compared to wood fibers, but are rich in lignin (25–30%) [43]. Cellulose is responsible for the strength of plant fibers; however, its low content in SCG limits the reinforcement potential of composites. Lignin, being a stiffer and more hydrophobic component, may slightly improve particle stiffness and compatibility with the matrix, but it is inherently brittle and does not form strong bonds with the polymer.

Cellulose and lignin fibers serve a stabilizing function in the composite; however, their presence is insufficient to provide adequate mechanical strength at the structural level. The limited adhesion of irregular SCG particles to the matrix and the structural discontinuity of the material, especially at high filler contents, lead to a reduced stress-bearing capacity and limited tensile strength.

Additionally, the fats and oils present in SCG can act as an internal lubricant—on one hand, they improve the wetting of particles by the polymer [26], but on the other hand, excessive amounts may cause particle aggregation and weaken the composite structure [44].

Comparing these results with those of composites with wood powder and hemp fibers, it is concluded that the use of wood powder allows obtaining significantly better mechanical properties than those of composites with SCG. Wood powder has a more homogeneous structure and better adhesion to the matrix, which allows for higher strength and stiffness. The modulus of elasticity of the composites with wood powder, which exceeded 6000 MPa, was higher than that of the SCG composites. This was attributed to the fine-grained and isotropic nature of the wood powder, which created a more coherent structure. Composites with hemp fibers, on the other hand, have the highest values of the modulus of elasticity and tensile strength, which is the effect of the presence of continuous, strong fibers with high mechanical strengths. Hemp fibers, acting as microreinforcements, can effectively transfer loads and limit deformations in the matrix, which is not possible when using irregular SCG.

### 3.5. Hardness Assessment

**Figure 4:**
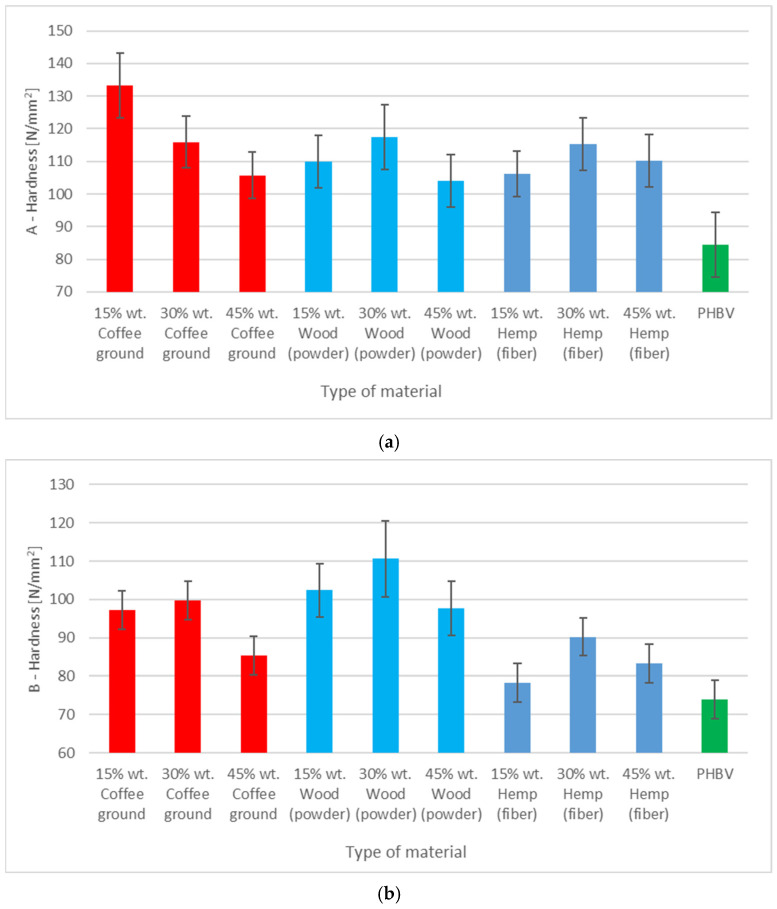
Hardness test results for different PHBV materials: (**a**) in A zone, (**b**) in B zone.

The highest hardness of all tested composites reaches the value of about 133 N/mm^2^ (Figure 4). An increase in the content of SCG to 30% and 45% caused a decrease in hardness to about 116 and 106 N/mm^2^, respectively. In comparison, the composites with wood powder showed a relatively stable hardness in the A region, regardless of the filler content, with values in the range of 104–117 N/mm^2^. Similar values and trends were observed for the biocomposites with hemp fibers. In contrast, the hardness value of pure PHBV in the A region was about 85 N/mm^2^, which indicated that the fillers increased the hardness of the composite in this region.

In area B, located at the ends of the sample, the hardness of the composite with coffee grounds is more even but slightly lower than that in area A. For 15% filler, it is about 97 N/mm^2^, and at 30% and 45%, it changes to about 100 and 85 N/mm^2^, respectively. In composites with wood powder, the hardness in area B was about 97–110 N/mm^2^, which confirmed their homogeneous structure. The composites with hemp fibers also showed the lowest hardness (in the group of biocomposites) in area B at about 78–90 N/mm^2^, which was near to the value for pure PHBV (about 75 N/mm^2^).

### 3.6. Impact Tensile Strength Assessment

**Figure 5:**
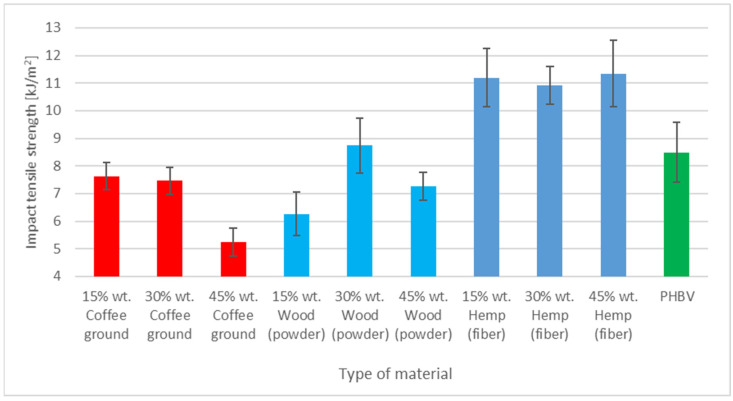
Impact tensile strength of PHBV composites with different fillers.

The coffee grounds composites were also analyzed in terms of their impact tensile strength and compared with those of other materials [7,31,32] (Figure 5). Pure PHBV exhibited an impact tensile strength of about 8 kJ/m^2^, which was considered the initial value.

For composites with SCG, a consistent trend was observed. The composite containing 15% coffee grounds by weight exhibited an impact strength (Figure 5) comparable to that of pure PHBV, with values around 7.5 kJ/m^2^. With an increase in the coffee ground content to 30%, the value showed only slight variation, and with a further increase to 45%, it gradually decreased to approximately 5 kJ/m^2^. This outcome may result from the unique morphological properties, structure, and chemical composition of SCG. Coffee grounds are characterized by irregular shapes and varied particle sizes, which affect their interactions with the polymer matrix. As the coffee grounds content increased to 45%, a reduction in the impact tensile strength was observed. This may be attributed to the higher filler content, which can lead to the formation of voids or microvoids, potentially weakening the composite structure. This is probably due to excessive filler, which introduces a large number of empty spaces or microvoids, thereby weakening the structure of the composite. Irregular coffee particles in large quantities can also result in the formation of clusters that act as weak spots in the matrix and are more susceptible to crack propagation during impact loading. A high ground content can also disrupt the homogeneity of the matrix, leading to local stress concentrations and reduced impact energy dissipation efficiency. As a result, although small amounts of SCG have a beneficial effect, their excess reduces the impact tensile strength of the composite.

In comparison, the composites with wood powder showed more stable impact tensile strength results, which were close to that of pure PHBV at 15% filler content and even increased to about 8.5 kJ/m^2^ at 30%. One possible reason for the relatively small reduction in the impact strength of the composite with wood flour (compared to less homogeneous fillers) may be the isotropic, fine-grained nature of this filler. Such wood flour is more easily dispersed uniformly within the matrix, forming a relatively continuous and homogeneous structure that promotes an even distribution of stress during impact [n]. However, similar to SCG, the higher content of wood powder (45%) began to weaken the structure, which may be the result of limited matrix cohesion at high filler content.

The highest impact strength values, reaching 10–11.5 kJ/m^2^, were achieved by composites with hemp fibers. These fibers act as effective micro-reinforcement and absorb impact energy by reducing stress. Due to the high mechanical strength of hemp fibers and their ability to evenly transfer loads, the composites with this filler are characterized by higher resistance to crack propagation. The observed higher impact strength values for the composite with hemp fibers may have resulted from better integration of these fibers with the matrix. Hemp fibers, which are long and rough, provide a more effective load transfer compared to irregular particles (e.g., spent coffee grounds) [7,32].

### 3.7. The Shrinkage Determination

**Figure 6:**
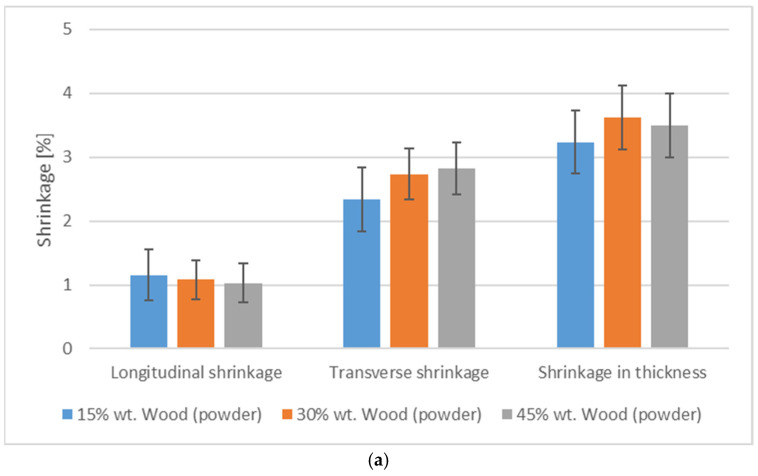

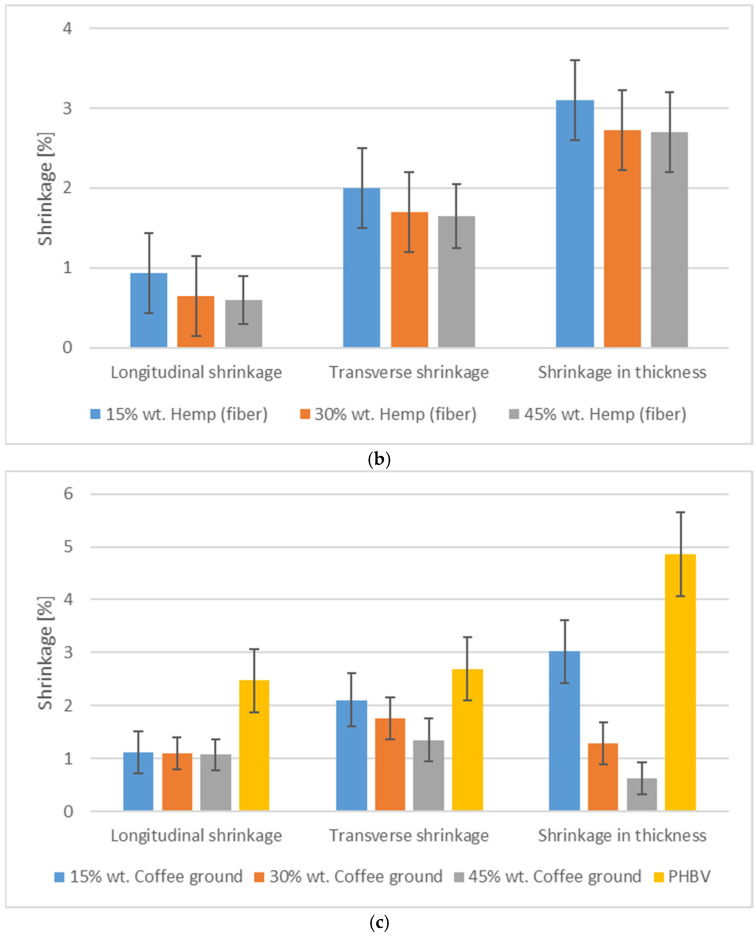
Summary of longitudinal, transverse, and thickness shrinkage values for various types of filler: (**a**)—wood flour, (**b**)—hemp fiber, (**c**)—coffee grounds, and pure PHBV.

The biocomposite shrinkage was also analyzed in the tests of the quality and shape dimensional accuracy of the obtained molded pieces (Figure 6). Based on the presented graph, it is possible to assess the shrinkage of composites containing different levels of spent coffee grounds filler tested in three directions: longitudinal, transverse, and thickness. In the case of longitudinal shrinkage, for composites with 15%, 30%, and 45% filler contents, the shrinkage values were similar and oscillated around 1%. An increase in the amount of coffee filler did not significantly affect the longitudinal shrinkage of the composite, which may suggest that in this direction, the composite structure is stable regardless of the SCG content. The transverse shrinkage for each filler content also remained at a similar level, at around 2 to 1.5%, with a minimal tendency to decrease at higher filler contents. Similar to the case of longitudinal shrinkage, an increase in the coffee ground content did not have a significant effect on the transverse shrinkage of the composite, which may mean that the coffee grounds did not significantly affect the matrix to limit the shrinkage in this direction. The situation is completely different in the case of shrinkage in thickness, where a clear decrease in the shrinkage value is noticeable with increasing filler content. For a composite with 15% SCG, the shrinkage in thickness was about 3%, while with 30% and 45% SCG content, these values decreased to about 1.3% and 0.5%, respectively. A significant decrease in shrinkage in thickness with a higher coffee ground content may indicate an increase in the structural stability of the composite in this direction, which may be the result of a more compact composite structure increasing resistance to shrinkage. SCG can act as a filler to counteract deformations in the thickness direction, while their effect in other directions remains limited, probably due to insufficient strength or adhesion to the matrix.

The analysis of the results indicates significant differences in the shrinkage of the various composites. Pure PHBV showed longitudinal shrinkage at a level of about 2.5%, while the composites with spent coffee grounds and wood powder were more stable in this direction, with values in the range of 1–2%. In turn, the use of increasing amounts of flax fibers reduced the longitudinal shrinkage to about 0.5%. In terms of transverse shrinkage, pure PHBV showed higher values (about 2.5%) than the composites with fillers, which remained at a level of about 1–3%. The addition of most of the analyzed fillers to the PHBV matrix stabilized the transverse shrinkage. The greatest differences were observed in the through-thickness shrinkage. Here, pure PHBV reached the highest value of about 5%, significantly exceeding the results of the composites with additives. The composites with SCG showed a clear reduction in shrinkage thickness, especially with a higher filler content. where the shrinkage value decreased to about 0.5%.

Filler geometry has a significant impact on the shrinkage value of composites since the shape, size, and proportions of the fillers affect the way the fillers interact with the polymer matrix. Different filler geometries, such as fibers, powders, and flakes, can affect shrinkage. Hemp fibers allow better stress transfer in one direction (most often, along their length). The fibers act as microreinforcements, which limit shrinkage mainly in the direction perpendicular to their orientation. In the case of composites with fibers, such as hemp fiber, we often observe greater dimensional stability in the longitudinal direction, but their effect on transverse and thickness shrinkage may be limited, especially if the fibers are not evenly distributed or do not have good adhesion to the matrix. Powders (e.g., wood powder) have an isotropic geometry. The properties were similar in all the directions. In the case of powders, such as wood powder, the shrinkage of the composite can be reduced more evenly in all directions because the powders form a more homogeneous structure. This allows the powders to effectively reduce both the through-thickness and transverse shrinkage, making them good fillers for the dimensional stabilization of composites. SCG are an unusual filler with an irregular shape that can act similarly to powder but with greater porosity and size inhomogeneity. This geometry causes spent coffee grounds to create micropores and a complex structure within the composite, effectively reducing the through-thickness shrinkage. Irregular shapes can affect shrinkage to varying degrees depending on their distribution and size; however, in thicker parts, they can create “shrinkage blocks” that reduce its value more effectively. Additionally, spent coffee grounds are rich in organic compounds, such as cellulose, lignin, hemicelluloses, oils, and fatty acids. Cellulose and lignin, due to their stiffness, can act as microreinforcements thatlimit the through-thickness shrinkage in the composite, especially as the ground content increases. Fatty acids and other organic compounds can improve the adhesion between the filler and the matrix. This increases the structural stability and helps reduce shrinkage, especially in the thickness direction. Due to their porous structure, SCG can also partially absorb moisture, which helps reduce internal stresses and shrinkage during composite cooling. At the same time, their irregular shape causes them to act uniformly in different directions, which helps achieve more comprehensive dimensional stabilization compared to unidirectional fibers. The orientation and proportion of the fillers also play important roles. Fibers have a greater effect on shrinkage when they are oriented parallel to the direction of external forces, which increases the shrinkage resistance in a given direction. Powders and irregular particles, on the other hand, are less susceptible to orientation, which can provide more uniform dimensional stability. Increasing the amount of fillers results in a greater contact between them, which increases the structural stability of the composite and reduces shrinkage, especially in the thickness direction. This was also observed in composites with spent coffee grounds and wood powder.

### 3.8. Microstructure Evaluation at Fractures

**Figure 7:**
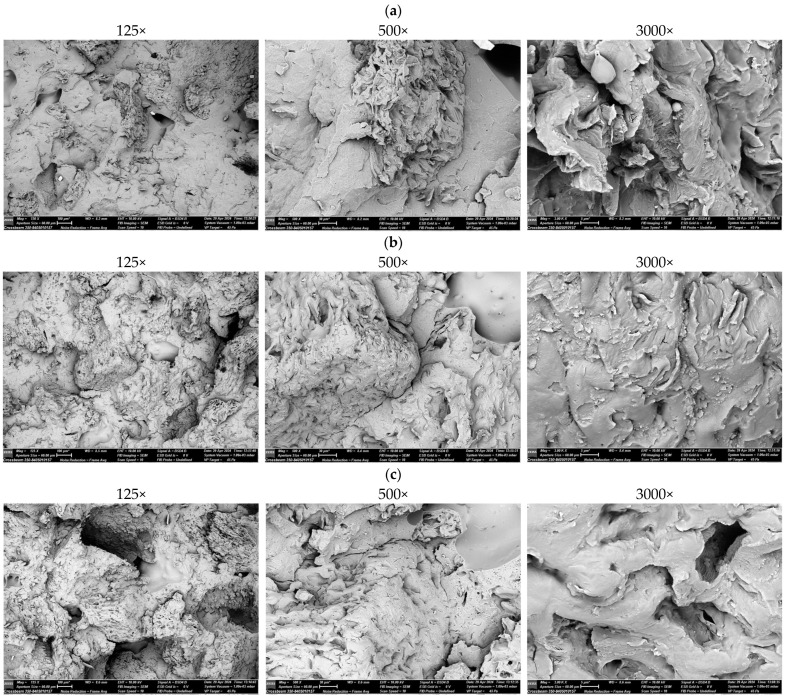
SEM micrographs of biocomposites at fractures containing: (**a**) 15 wt.% coffee grounds, (**b**) 30 wt.% coffee grounds, (**c**) 45 wt.% coffee grounds.

When analyzing the SEM micrographs of the PHBV composite with a filler in the form of ground SCG with a content of 15 wt% (Figure 7a), the details of the material structure were visible at different magnification levels. A magnification of 125× shows the general structure of the composite after the uniaxial stretching. Cracks and structural separations were observed. These are probably places where the filler (coffee particles) separated from the PHBV polymer matrix, which is typical for fractures with low adhesion between the matrix and filler. Large cavities and voids (which may be the result of microscale pores or pulled-out filler particles) are also visible, suggesting that the distribution of coffee particles may be uneven at this scale. At a higher magnification (500×), the structure began to reveal more detailed features of the fracture. A porous structure is visible, which is most likely related to the presence and distribution of coffee particles in the polymer matrix. The fracture edges appear irregular, indicating the fragility of the composite matrix. It is also possible that the visible layers are interfacial fractures between the filler and the PHBV matrix, which may indicate poor interfacial interaction. At the highest magnification (3000×), more distinct structural features of the fracture surface can be observed. Micropores and fine fibers or frays were visible on the surface, which may be due to the differences in the mechanical properties of the filler and PHBV. The surface appears inhomogeneous, confirming the previous assumptions about the limited adhesion between the filler and matrix. The structure also appeared to have clear cracks, which may indicate fragility and low tensile strength in the presence of SCG as a filler. All three SEM micrographs suggest that the PHBV composite with spent coffee grounds filler shows some fragility and poor interaction between the polymer matrix and the filler. Coffee particles were not fully integrated into the matrix, which can lead to early cracking and failure under mechanical stress.

SEM micrographs show fractures of the PHBV polymer composite with 30% by mass of ground SCG (Figure 7b). At the lowest magnification (125×) a highly jagged fracture morphology of the composite is visible, which shows numerous inhomogeneities. The surface was strongly corrugated with distinct pores and spaces. The presence of numerous large voids suggests that a higher proportion of coffee particles (30%) causes a weaker bond between the polymer phase and the filler. This type of porosity indicates that some coffee particles may have been torn out of the matrix during stretching, resulting in the formation of local voids. Magnification of 500× revealed a more detailed fracture texture. It could be observed that the coffee particles were non-uniformly dispersed, and the fracture surface becomes rougher and irregular. It seems that a higher filler content leads to larger coffee clusters, which may act as defects in the composite structure, facilitating its fracture. The fracture edges were irregular, which may indicate local stresses occurring at the boundary of the polymer phase and filler. At maximum magnification (3000×), the fracture surface revealed even more details. Clear micropores and cracks were visible in the polymer matrix, which may have resulted from the poor compatibility between the filler and PHBV. The structure appeared jagged and irregular, which suggested that the increased amount of filler contributed to the brittleness of the material. Small flakes and irregularities were also observed. This could have been caused by an uneven stress distribution during the tensile test. SEM micrographs show that the PHBV composite with 30% ground coffee grounds exhibits clear features related to brittleness and weakened matrix cohesion. Higher coffee content promoted the formation of numerous voids and spaces, which weakened the integrity of the material. The irregularity of the structure visible in the images suggests that a higher filler concentration negatively affects the adhesion between the polymer phase and filler.

SEM micrographs (Figure 7c) illustrate the fracture structure of the PHBV polymer composite with 45% coffee grounds by mass. Magnification 125× shows a clearly rough and inhomogeneous structure of the composite. Large pores and numerous voids are visible, which may indicate the detachment of coffee particles from the PHBV matrix due to stress. The high filler level (45%) made the composite appear more porous and less cohesive, which may be due to insufficient compatibility between the polymer phase and SCG. Such a large amount of filler implied that the spaces between the coffee particles were not effectively filled with the polymer, which resulted in the formation of clear gaps in the structure. At 500× magnification, a more detailed texture of the material was observed. Irregularities and porosity were visible, which indicated a weakening of the cohesion of the composite. The coffee particles appeared to be irregularly dispersed and formed clusters. These filler densities act as structural defects that promote crack propagation. The PHBV matrix appeared to have a limited ability to encapsulate coffee particles, resulting in the formation of irregular fractures with weakly bonded boundaries. At 3000× magnification, microscale porosities and numerous edges and microcracks are visible in the composite structure, suggesting brittleness caused by the large amount of filler. With such a high content of coffee grounds, the material did not show sufficient adhesion between the filler and the polymer matrix, leading to fragmentation and flaking at the microscopic level. The fracture surface was jagged and full of small crevices, indicating the poor mechanical properties of this composite. All three SEM micrographs clearly show that the PHBV composite with 45 wt.% coffee grounds is characterized by very poor structural cohesion. However, such a high filler content significantly weakens the material, leading to numerous structural defects, such as pores, voids, and irregular fractures. The limited phase compatibility between PHBV and spent coffee grounds at this filler content caused the composite to be brittle and crack easily under load.

When analyzing the SEM micrographs of the PHBV composites with spent coffee grounds contents of 15%, 30%, and 45%, clear differences in the microstructure were observed, which had a significant impact on the mechanical properties of the material. An increase in the filler content leads to a change in the fracture morphology and structural cohesion of the composites, which, in turn, affects their resistance to mechanical loads and overall strength. When analyzing the composite containing 15% filler in the context of SEM micrographs and mechanical properties, it can be concluded that, due to better adhesion between the filler and the polymer matrix, this composite should be characterized by higher strength and greater tensile strength. The PHBV matrix effectively transferred stress to the coffee particles, which strengthens the material and minimizing the risk of crack propagation. In turn, for the composites containing 30% filler, such a structure resulted in a decrease in the mechanical strength of the composite. Coffee grounds clusters acted as stress concentration points that promoted the initiation and propagation of cracks under load. The composite became more brittle, and the matrix was no longer able to effectively transfer stress to the coffee particles. In turn, for composites containing 45% filler, such a structure drastically reduces the mechanical properties of the material. The composite became extremely brittle and susceptible to cracking under load. A large number of voids and irregularities indicates that stresses cannot be effectively distributed in the material, which leads to rapid crack propagation. The mechanical strength of this composite was the lowest among all the analyzed samples.

The thermal properties were also affected by the microstructure. SCG acted as nucleating agents, slightly altering the crystallinity and thermal behavior of the polymer matrix. However, uneven filler dispersion caused localized variations in the thermal conductivity, particularly at higher filler contents. Increased porosity and clustering further disrupted thermal stability, reducing the effectiveness of the composite in thermal applications. This analysis highlights that achieving a homogeneous microstructure and improving filler-matrix bonding are critical for optimizing both the mechanical and thermal properties. Without addressing these challenges, higher filler contents lead to significant structural and property degradations.

## 4. Conclusions

In the extrusion process of the PHBV-SCG biocomposite, a trend related to the need to lower the temperatures of the extruder heating zones along with increasing the amount of filler was observed. These changes were necessitated by the need to maintain the proper viscosity (quality) of the extrudate. A similar trend was also observed in the injection molding process. The need to lower the processing temperatures may be related to the high heat capacity of coffee. This allows for the long-term accumulation of heat in the plasticized mass of a material.

The mechanical properties of the PHBV-SCG biocomposite were assessed. Deterioration of mechanical properties was found in relation to other fillers with increasing filler content, taking into account hardness, impact strength, and properties determined in the uniaxial tensile test. This is most likely due to the fact that the spent coffee grounds does not have a regular longitudinal geometry. Moreover, the morphology of SCG differs significantly from that of other natural fillers. Low cellulose content and elevated lignin content may result in decreased adhesion at the matrix-inclusion interface. A high proportion of lignin, on the other hand, can reduce the resistance to flow of the polymer in the cavity.

In the case of composites with natural fillers, especially those with fibrous geometries, there is a noticeable trend related to the increase in tensile strength to a certain amount of filler, beyond which these properties deteriorate. However, when using spent coffee grounds, the tensile strength decreased with increasing filler content in the PHBV matrix.

The modulus of elasticity increased for samples with up to 15% filler content, while it decreased with a further increase in the filler content. At the same time, it was observed that the increase in the filler amount increased the content of the crystalline phase in the polymer matrix, which is indicated by the increase in the absolute value of the melting enthalpy (for samples with 0, 15, 30, and 45% coffee filler content, it was 71.1, 79.0, 91.6, and 113.4 J/g, respectively). The nature of the changes in the modulus in connection with the composition and morphology of the samples indicates that the dominant effect on the value of the modulus is the filler addition, a large amount of which lowers the modulus. It is also possible to change the crystalline form of the polymer; for example, a large amount of filler may result in the formation of a large number of fine crystallites.

The quality and dimensional accuracy of the samples showed that the shrinkage of the products manufactured from PHBV filled with coffee grounds was significantly reduced along with the increase in the amount of this filler. This was especially evident in the case of shrinkage in the thickness of the part. This trend may be related to the chemical composition of the coffee. Low cellulose content and high lignin content may result in lower resistance to the flow of the polymer, which results in better and easier filling of the mold cavity.

It is worth noting that the use of SCG as a filler in the biodegradable PHBV matrix aligns with the concept of waste management and the circular economy, replacing part of the polymer raw material with a naturally derived material. When using this type of filler at 45 wt.%. plastic production costs can be reduced by approx. 45% compared to pure PHBV. This may also allow faster and wider commercialization possibilities for this type of biocomposite in injection molded products.

## Figures and Tables

**Table 1:** Temperatures of extruder heating zones for composites with different types of natural fillers (based on own research and [7,31,32]).

Type of Filler	Head [°C]	Zone 3 [°C]	Zone 2 [°C]	Zone 1 [°C]	Feed Hopper Zone [°C]	Screw Speed [rpm]
PHBV	160	160	155	145	50	100
**15% wt. filler content**						
Coffee ground	175	170	160	150	35	100
Wood (powder)	164	162	160	154	31	80
Hemp (fiber)	170	165	155	145	35	100
**30% wt. filler content**						
Coffee ground	165	160	160	155	35	100
Wood (powder)	175	170	160	150	35	80
Hemp (fiber)	175	170	160	150	35	100
**45% wt. filler content**						
Coffee ground	157	155	150	148	33	100
Wood (powder)	180	175	165	155	30	100
Hemp (fiber)	180	175	165	155	35	100

**Table 2:** The processing temperatures for composites with different types of natural fillers (based on own research and [7,31,32]).

Type of Filler	Melt Temperature	Mold Temperature
PHBV	167	60
**15% wt. filler content**		
Coffee ground	183	80
Wood (powder)	170	65
hemp (fiber)	167	60
**30% wt. filler content**		
Coffee ground	175	80
Wood (powder)	175	85
hemp (fiber)	185	85
**45% wt. filler content**		
Coffee ground	170	80
Wood (powder)	180	90
hemp (fiber)	190	90

**Table 3:** Common processing parameters for all types of fillers used.

Parameter	Value
Cooling time [s]	20
Packing time [s]	25
Packing pressure [MPa]	30
Injection speed [cm^3^/s]	35

**Table 4:** Parameters and enthalpy of thermal processes recorded during heating of analyzed samples determined by DSC.

Sample	T_onset_ [°C]	T_min_, Peak1 [°C]	T_min_, Peak2 [°C]	Enthalpy of Whole Endothermic Process [J/g]	Corrected Enthalpy of the Endothermic Process [J/g]
**PHBV** **(Sample 0)**	161.6	-	176.5	−71.1	−71.1
**15% wt. filler** **(Sample I)**	160.3	168.1	176.1	−67.2	−79.0
**30% wt. filler** **(Sample II)**	161.3	-	173.9	−64.1	−91.6
**45% wt. filler** **(Sample III)**	160.4	173.1	176.8	62.6	−113.4

**Table 5:** Comparison of the results of static tensile tests for PHBV-coffee grounds composite with PHBV composites with other fillers.

Type of Filler	E [MPa]	σ [MPa]	ε_m_ [%]
PHBV (x *)	2617.37	35.48	4.12
s **	112.02	0.86	0.15
15% wt. coffee ground (x)	3308.81	23.91	1.14
s	115.16	2.31	0.55
30% wt. coffee ground (x)	3085.36	20.65	1.33
s	22.43	0.60	0.23
45% wt. coffee ground (x)	3092.54	16.54	1.28
s	36.79	0.21	0.02
15% wt. Wood (powder) (x)	5459.58	31.11	0.90
s	147.16	1.68	0.12
30% wt. Wood (powder) (x)	6022.56	30.48	1.07
s	414.56	0.73	0.06
45% wt. Wood (powder) (x)	6060.63	28.07	1.07
s	985.43	0.29	0.02
15% wt. Hemp (fiber) (x)	5242.29	39.03	2.68
s	196.29	0.59	0.17
30% wt. Hemp (fiber) (x)	6992.31	42.90	2.28
s	199.44	0.71	0.06
45% wt. Hemp (fiber) (x)	7162.28	25.47	0.57
s	1006.93	6.73	0.30

* x—the average value. ** s—standard deviation.

## Data Availability

The original contributions presented in this study are included in the article. Further inquiries can be directed to the corresponding author.
